# MODOMICS: a database of RNA modifications and related information. 2025 update and 20th anniversary

**DOI:** 10.1093/nar/gkaf1284

**Published:** 2025-11-24

**Authors:** Dominik Sordyl, Etienne Boileau, Agata Bernat, Satyabrata Maiti, Sunandan Mukherjee, S Naeim Moafinejad, Masoud Amiri Farsani, Anastasiya Shavina, Andrea Cappannini, Giada Agostini, Silvestro G Conticello, Filip Stefaniak, Christoph Dieterich, Elżbieta Purta, Janusz M Bujnicki

**Affiliations:** Laboratory of Bioinformatics and Protein Engineering, International Institute of Molecular and Cell Biology in Warsaw, ul. Ks. Trojdena 4, PL-02-109 Warsaw, Poland; Klaus Tschira Institute for Integrative Computational Cardiology, Im Neuenheimer Feld 669, 69120 Heidelberg, Germany; Department of Internal Medicine III, University Hospital Heidelberg, Im Neuenheimer Feld 410, 69120 Heidelberg, Germany; German Center for Cardiovascular Research–Partner site Heidelberg/Mannheim, Im Neuenheimer Feld 669, 69120 Heidelberg, Germany; Laboratory of Bioinformatics and Protein Engineering, International Institute of Molecular and Cell Biology in Warsaw, ul. Ks. Trojdena 4, PL-02-109 Warsaw, Poland; Laboratory of Bioinformatics and Protein Engineering, International Institute of Molecular and Cell Biology in Warsaw, ul. Ks. Trojdena 4, PL-02-109 Warsaw, Poland; Laboratory of Bioinformatics and Protein Engineering, International Institute of Molecular and Cell Biology in Warsaw, ul. Ks. Trojdena 4, PL-02-109 Warsaw, Poland; Laboratory of Bioinformatics and Protein Engineering, International Institute of Molecular and Cell Biology in Warsaw, ul. Ks. Trojdena 4, PL-02-109 Warsaw, Poland; Laboratory of Bioinformatics and Protein Engineering, International Institute of Molecular and Cell Biology in Warsaw, ul. Ks. Trojdena 4, PL-02-109 Warsaw, Poland; Laboratory of Bioinformatics and Protein Engineering, International Institute of Molecular and Cell Biology in Warsaw, ul. Ks. Trojdena 4, PL-02-109 Warsaw, Poland; Laboratory of Bioinformatics and Protein Engineering, International Institute of Molecular and Cell Biology in Warsaw, ul. Ks. Trojdena 4, PL-02-109 Warsaw, Poland; Institute of Clinical Physiology, National Research Council, Via Pieraccini 6, 50139 Firenze, Italy; Department of Medical Biotechnologies, Università di Siena, Viale Mario Bracci 16, 53100 Siena, Italy; Institute of Clinical Physiology, National Research Council, Via Pieraccini 6, 50139 Firenze, Italy; Core Research Laboratory, ISPRO-Institute for Cancer Research, Prevention and Clinical Network, 50139 Firenze, Italy; Laboratory of Bioinformatics and Protein Engineering, International Institute of Molecular and Cell Biology in Warsaw, ul. Ks. Trojdena 4, PL-02-109 Warsaw, Poland; Klaus Tschira Institute for Integrative Computational Cardiology, Im Neuenheimer Feld 669, 69120 Heidelberg, Germany; Department of Internal Medicine III, University Hospital Heidelberg, Im Neuenheimer Feld 410, 69120 Heidelberg, Germany; German Center for Cardiovascular Research–Partner site Heidelberg/Mannheim, Im Neuenheimer Feld 669, 69120 Heidelberg, Germany; Laboratory of Bioinformatics and Protein Engineering, International Institute of Molecular and Cell Biology in Warsaw, ul. Ks. Trojdena 4, PL-02-109 Warsaw, Poland; Laboratory of Bioinformatics and Protein Engineering, International Institute of Molecular and Cell Biology in Warsaw, ul. Ks. Trojdena 4, PL-02-109 Warsaw, Poland

## Abstract

MODOMICS is the reference database of RNA modifications and related information, integrating chemical, biochemical, structural, and functional data. In this 2025 update, marking the 20th anniversary of MODOMICS, the database has been significantly expanded in scope and depth. The RNA sequence section now includes transcriptome-wide data generated in collaboration with Sci-ModoM, a quantitative resource of high-throughput epitranscriptomic datasets, enabling the representation of >48 000 transcripts with high-confidence modification annotations. The catalog of modified residues was improved with systematic curation, and addition of new computational descriptors derived from quantum mechanical calculations. An all-versus-all similarity analysis of chemical structures was performed, and the results are provided as an interactive similarity graph to explore chemical relationships among modifications. The protein section has been updated with newly characterized enzymes and expanded annotations of modification pathways, supported by an improved evidence and reliability framework. Together, these advances further strengthen MODOMICS as a comprehensive and reliable community resource, serving as both a reference and a platform for discovery in the rapidly developing field of epitranscriptomics. MODOMICS is available at https://iimcb.genesilico.pl/modomics/.

## Graphical Abstract

**Graphical Abstract:**
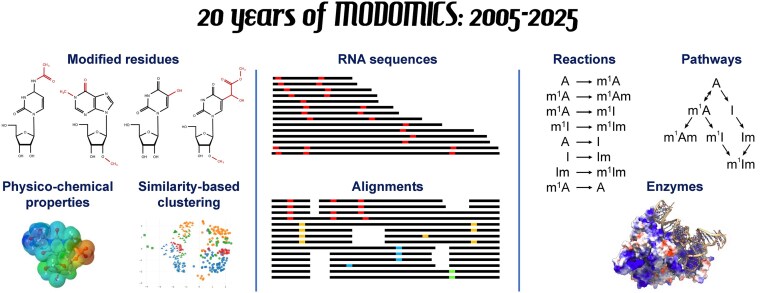

## Introduction

RNA modifications constitute a fundamental layer of gene expression regulation and cellular physiology. Beyond the four canonical ribonucleotides, >170 chemically distinct modifications have been identified in all classes of RNA molecules, including transfer RNAs (tRNAs), ribosomal RNAs, messenger RNAs (mRNAs), and diverse noncoding RNAs [1]. These modifications influence RNA folding, stability, localization, and translation. For some of them, these effects are mediated by specialized protein readers, writers, and erasers [2]. Certain modifications can also participate in biosynthetic pathways that yield hypermodified residues and, in some cases, are reversible, thereby enabling dynamic responses to cellular and environmental cues [3].

The study of RNA modifications has expanded dramatically over the last two decades, driven by methodological advances such as high-throughput sequencing, mass spectrometry, and more recently, nanopore-based detection [4–6]. High-resolution cryo-electron microscopy (cryo-EM) has further revolutionized the field, allowing direct visualization and assignment of modified residues within ribosomes [7]. Our recent work has combined cryo-EM and computational modeling to investigate the structural consequences of pseudouridine incorporation in human tRNAs, revealing how specific modifications influence RNA conformation and stability [8]. Alongside naturally occurring modifications, synthetic alterations have been deliberately introduced into RNAs for structural, functional, and therapeutic purposes, broadening the scope of RNA chemistry [9, 10]. The biological and biomedical relevance of RNA modifications has become increasingly clear, with links to cancer, cardiac, metabolic and neurological disorders, host–pathogen interactions, and the design of RNA-based drugs and vaccines [11–13].

MODOMICS was created in 2005 as the first comprehensive resource devoted to RNA modifications, their chemical structures, biosynthetic pathways, enzymes, and localization in RNA sequences [14]. The idea originated from discussions between Janusz Bujnicki and Henri Grosjean, with inspiration drawn from REBASE, the database of DNA restriction–modification systems [15]. At that time, the greater diversity of methylated ribonucleosides, compared to the few known DNA modifications, motivated the creation of a resource covering RNA modification enzymes but also for pathways, especially those leading to hypermodifications. The first MODOMICS version was modest but quickly attracted a broad user base, and successive updates expanded the database into a central hub for the RNA modification community [16–19]. Key previous extensions included a catalog of building blocks for chemical synthesis, mass spectrometry and chromatography data, and links between modifications and disease. Despite limited dedicated funding, MODOMICS has persisted for two decades, and its recent inclusion in the IN-MOL-CELL research infrastructure at the International Institute of Molecular and Cell Biology provides hope for improved sustainability.

## Materials and methods

MODOMICS continues to be implemented as a relational database with a Python/Django backend, using SQLite as the primary database engine, and a JavaScript-based frontend. The system provides both a graphical web interface and a programmatic REST API, supporting standard output formats such as CSV and JSON. In this update, interactive visualization of chemical similarity data was implemented using web-based technologies including Plotly.js (https://plot.ly) for interactive scatter plot generation, RDKit.js (https://www.rdkit.org) for client-side molecular structure rendering, and Papa Parse (https://www.papaparse.com) for CSV data processing. The visualization interface allows users to select between different dimensionality reduction results (PCA, t-SNE, UMAP, Isomap, Kernel PCA, LLE, MDS, and Spectral Embedding) and displays chemical modifications as interactive points colored by reference nucleobase and shaped by moiety type. The molecular Morgan fingerprints were calculated using the rdkit Python module and the decomposition was performed using scikit-learn (https://scikit-learn.org/stable).

### Database content

In continuity with past updates, MODOMICS hosts a catalog of modified residues, enzymes and guide RNAs responsible for individual reactions, RNA modification pathways, sequences of modified RNAs, a catalog of “building blocks” for chemical synthesis of modified RNA, links of RNA modifications to different diseases, and other associated data such as relevant publications. The catalog of modified residues includes both modified nucleoside and nucleotide residues occurring naturally, and synthetic modifications, especially those found in RNA structures determined experimentally and available in the RCSB PDB[20]. The MODOMICS nomenclature system enables unique encoding of different types of modifications, facilitating both human and computational interpretation of the data.

In response to user requests, MODOMICS now provides the main data types, particularly proteins involved in modifications and modified residues, as complete downloadable tables containing nearly all information available in individual records, with the exception of data presented as images, metafiles, or external links.

### Updated RNA sequence section

The key expansion in the current release of MODOMICS is the integration of transcriptome-wide high-throughput datasets. Until now, the sequences deposited in MODOMICS largely originated from detailed studies of individual molecules, in which modifications were annotated site by site. More recently, sequences of experimentally determined RNAs from the RCSB PDB were added, enabling the representation of modifications observed in structural studies. While these sequences remain invaluable, they represent only a small fraction of the epitranscriptome, which encompasses tens of thousands of transcripts in human cells. Until now, MODOMICS has not included the major transcript categories, in particular mRNAs and long noncoding RNAs (lncRNAs).

To address this limitation, we established a collaboration with Sci-ModoM, a newly developed quantitative database of transcriptome-wide high-throughput RNA modification sites [21]. Sci-ModoM integrates results from a wide variety of sequencing-based detection methods, representing >6 million modifications across 156 datasets, underpinned by the bedRMod format (https://dieterich-lab.github.io/euf-specs). Per-site and per-dataset information includes stoichiometry, coverage, and score, but Sci-ModoM does not provide RNA sequences explicitly. Our collaboration enabled the generation of annotated transcript sequences with consensus sets of modification sites.

Briefly, evidence (modification sites and associated metadata) was extracted from Sci-ModoM for all human autosomal chromosomes. Only single-resolution modifications were used, i.e. any evidence with a context site was discarded. Only datasets in Sci-ModoM that were supported by publicly available primary data were used. Annotations, assembly information, and genome sequences were downloaded from Ensembl, using release 110. Modification sites were then mapped to Ensembl transcript models. Transcript abundance was estimated with Salmon for every matching transcript where at least one modification was reported, for every available dataset. Finally, stringent selection criteria were applied. Transcripts were included only if they were supported by at least two datasets with expression levels above 0.75 Transcripts per Million (TPM). For each modification site, a minimum of two datasets had to provide consistent evidence with coverage of at least 10 and modification frequency of at least 50%. If a transcript contained at least one high-confidence modification site passing these filters, the transcript sequence was incorporated into MODOMICS. Sites that did not meet these criteria were excluded from the MODOMICS representation, meaning that only modifications with high confidence annotations remain marked as modified residues, while other positions are shown as unmodified.

This curation resulted in the addition of 48 836 transcripts to MODOMICS, of which 46 766 correspond to protein-coding genes, 1826 to lncRNAs, and smaller subsets to pseudogenes, small RNAs, and other RNA types. The detailed distribution of RNA types in the newly added sequences is provided in Supplementary Table S1. This constitutes the largest single expansion of the RNA sequence section in the history of MODOMICS, compared to 2136 sequences in the previous release. Notably, this marks the first inclusion of coding RNA and lncRNA sequences in MODOMICS, reflecting the growing importance of modifications of long RNA molecules in epitranscriptomic studies. In parallel, in collaboration with RNAcentral, MODOMICS now also exports noncoding RNA sequences for integration with other community resources [22]. Through collaboration with Sci-ModoM and RNAcentral, MODOMICS contributes to the FAIRification of epitranscriptomic data, promoting interoperability, traceability, and standardized representation across resources.

To further contribute to the epitranscriptomics field and assist researchers in generating new RNA sequence data with modifications, MODOMICS now includes a new section “Direct RNA sequencing” with a table summarizing software tools and basecallers developed for Oxford Nanopore direct RNA sequencing. The table provides links to GitHub repositories and literature references, and specifies the compatible flowcells (RNA002 and/or RNA004), enabling users to quickly identify relevant resources for the analysis of nanopore data in the context of RNA modification studies.

### Updated modifications section

**Figure 1.**
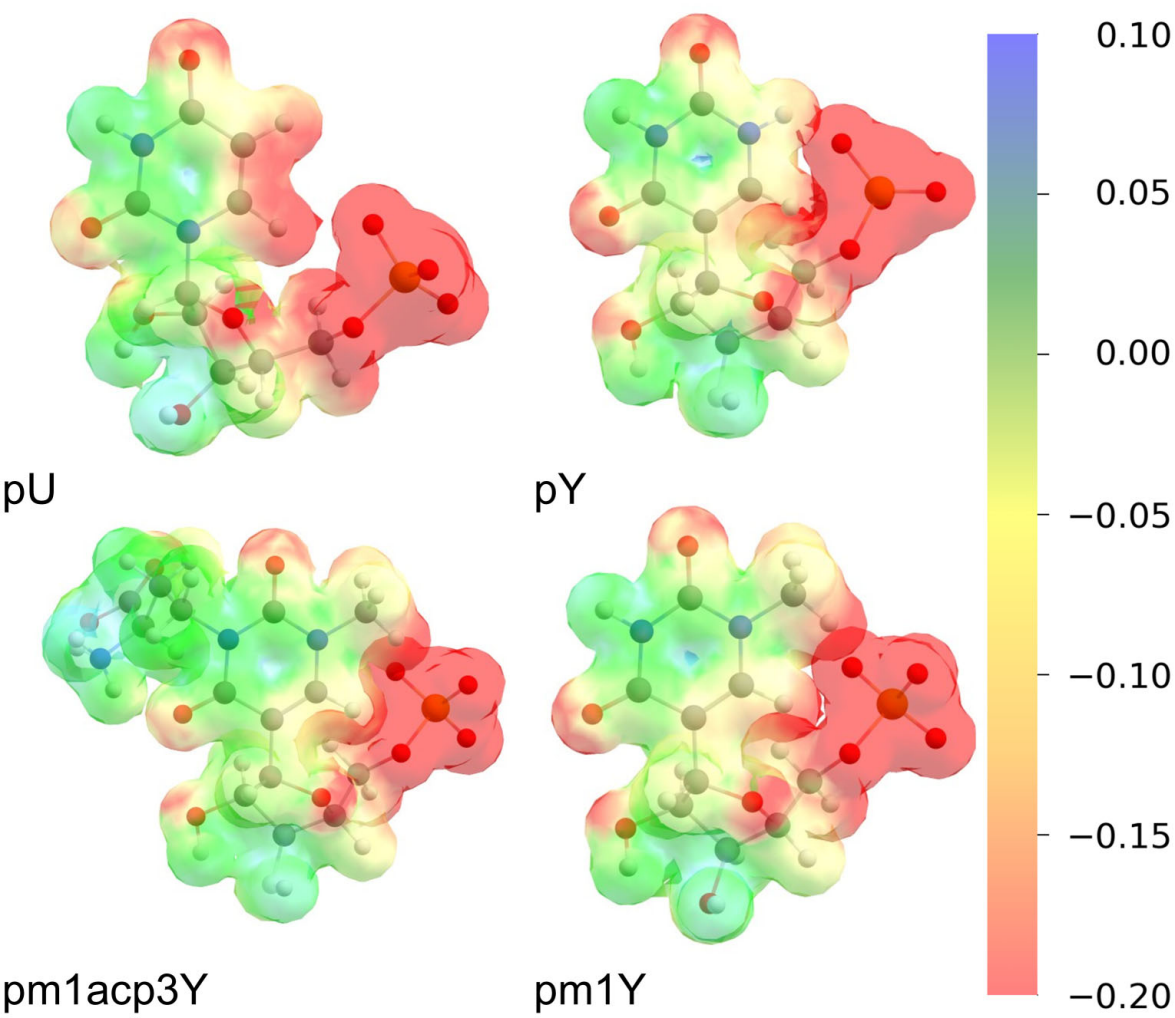
Electrostatic potential maps calculated for QM-optimized structures of nucleotides: uridine-5′-monophosphate (pU), pseudouridine-5′-monophosphate (pY), 1-methylpseudouridine-5′-monophosphate (pm1Y), and 1-methyl-3-(3-amino-3-carboxypropyl)pseudouridine-5′-monophosphate (pm1acp3Y). The electrostatic potential is mapped onto the electron density isosurface. The color scale represents the potential value, ranging from negative (red) to positive (blue), with neutral regions shown in green. The unit of the color bar is Hartree/e.

In this update, we curated the information on modified residues, especially with respect to biologically oriented residue names and chemically oriented IUPAC codes. We have also updated the atom nomenclature in the 3D models of modified residues that do not have the corresponding LIG entries in the RCSB PDB. The dataset of modified residues in MODOMICS was also expanded in the depth of chemical annotation. In the current release, all nucleoside and nucleotide residues were subjected to conformational optimization using a hybrid density functional method ωB97X-D3BJ/def2-SVPD level of theory [23] with the RIJCOSX approximation [24, 25] and the def2/J auxiliary basis set [26] with ORCA 6.1 quantum chemistry package [27]. For each residue, we provide energy-minimized geometries together with physicochemical descriptors that were derived directly from quantum mechanical (QM) calculations, including Mulliken atomic charges, dipole moments, the HOMO (highest occupied molecular orbital) and LUMO (lowest unoccupied molecular orbital) energy gap, and molecular electrostatic potential maps (examples illustrated in Fig. 1). The HOMO–LUMO energy gap relates to the electronic transitions that underlie UV absorption, providing insight into how nucleotide modifications can influence the photophysical properties and reactivity. These features offer a consistent and theory-based representation of modified residues. The QM-derived data are provided for download in standard file formats, ensuring their usability for downstream applications in molecular modeling and simulation studies. By providing optimized three-dimensional structures and reliable electronic parameters, MODOMICS facilitates the integration of RNA modifications into biophysical and computational workflows, including docking, molecular dynamics, and free energy calculations.

**Figure 2.**
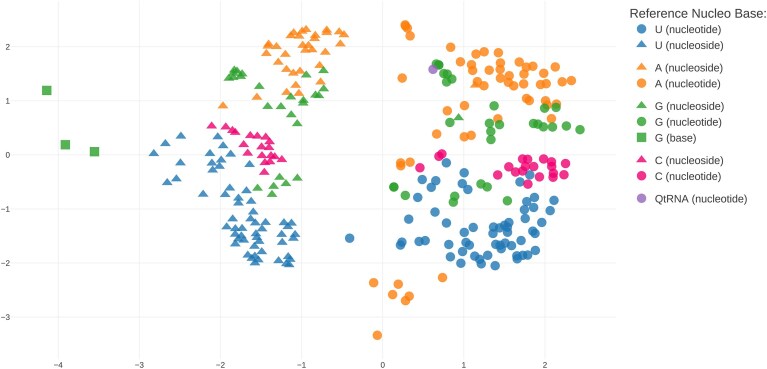
Visualization of chemical similarity between all nucleoside/nucleotide residues and modified free bases in MODOMICS (Tanimoto similarity of Morgan fingerprints) mapped to 2D space using the multidimensional scaling procedure.

In parallel, we performed an all-versus-all comparison of the chemical structures of modified residues present in MODOMICS using molecular fingerprints and Tanimoto similarity scoring. To visualize the high-dimensional similarity relationships, we applied dimensionality reduction techniques including PCA, t-SNE, UMAP, Isomap, Kernel PCA, LLE, MDS, and Spectral Embedding to map the similarity data into 2D coordinate space. The results are presented as an interactive 2D scatter plot visualization where each point represents a modified residue, colored by its reference nucleobase (A, U, G, C, or QtRNA), and shaped according to its moiety type (Fig. 2). Users can hover over points to view the modification name and chemical structure, and click on any point to directly access the corresponding modification record in MODOMICS. This interactive visualization enables users to explore chemical relationships between modifications, identify clusters of structurally related residues, and understand how chemical diversity is distributed across the modification landscape. This representation provides a valuable resource for exploring the chemical space of RNA modifications.

### Updated proteins section

The MODOMICS section on proteins involved in RNA modification pathways was updated to include 101 new proteins involved in RNA modification reactions. Annotations of various protein entries were updated based on the literature review. In the current update, we continued to expand and refine the annotation of MODOMICS entries using the evidence and reliability scheme that had been introduced on a limited scale in the previous release. Particular effort was directed toward the section on enzymes involved in RNA modification reactions, where reliability scores were adjusted to reflect the confidence supported by the available data. The annotation framework helped to distinguish clearly whether an enzyme’s function is supported by direct biochemical characterization or by indirect evidence. In our categorization scheme, direct evidence requires a demonstration that an enzyme introduces a specific modification at a defined position in a particular transcript substrate. In contrast, the absence of a modification in a mutant strain lacking the corresponding gene is considered indirect evidence. For many enzymes, annotations were updated to incorporate recent experimental findings and literature. As in previous editions of MODOMICS, we minimized the inclusion of information predicted purely computationally and, for example, we generally excluded proteins assigned functions only on the basis of evolutionary similarity to experimentally characterized homologs. This systematic curation further improves the transparency and usability of MODOMICS, ensuring that users can readily assess the robustness of information concerning RNA-modifying enzymes and their pathways. Our ongoing goal is to expand this annotation framework to all data categories. At present, any entries not yet annotated are labeled under evidence and reliability class 5, signifying pending annotation and assessment. As always, MODOMICS users are encouraged to provide feedback to enhance the quality of these annotations.

### Discussion and future prospects

The present article marks the 20th anniversary of MODOMICS, which provides an opportunity to reflect on its trajectory and its contribution to the maturation of RNA modification research from a niche topic into a central component of molecular biology. Over two decades, MODOMICS has transformed from a manually curated catalog of chemical structures and pathways into a comprehensive, interoperable platform that integrates structural, biochemical, and functional information about modified residues in RNA molecules. Each successive update has addressed the evolving needs of the community by expanding the scope of the database, introducing standardized nomenclature, linking to external resources, and enhancing annotation systems.

The current release illustrates how MODOMICS can serve as a bridge between high-confidence, curated knowledge and the rapidly growing body of high-throughput epitranscriptomic data. The integration with Sci-ModoM provides a concrete example of this direction, combining transcriptome-wide evidence with robust annotation schemes to balance completeness with reliability. This synergy reflects a broader movement within the community, as embodied by initiatives such as the Human RNome Project, which aim to establish comprehensive reference datasets that capture both canonical sequences and their chemical modifications [28].

By adding QM-derived parameters and a similarity graph, MODOMICS now allows direct comparison of modified nucleosides based on their chemical properties. These features make it possible to quantify structural differences, and identify related chemical moieties.

Looking forward, MODOMICS will continue to expand along several complementary directions. First, systematic annotation of evidence and reliability will be extended to cover all data categories, including modifications in coding and noncoding RNAs, enzymes, and pathways. Second, reciprocal links with Sci-ModoM and other community resources will be strengthened, fostering an ecosystem of interoperable databases that collectively provide both depth and breadth of information. Third, the growing interest in RNA-based therapeutics highlights the importance of integrating data on synthetic modifications. In particular, we plan to extend the scope of the unnatural modifications section to include nucleotide- and nucleobase-based drugs, as well as modified residues used in functional studies, beyond those currently present in the RCSB PDB. MODOMICS will continue to adopt FAIR data principles to ensure that its content remains accessible, reusable, and compatible with emerging standards in the epitranscriptomics field.

We plan to strengthen MODOMICS sustainability and data management practices in the coming years. Depending on available resources, we intend to introduce dataset versioning and increase the update frequency from biannual to more regular releases, ideally on a quarterly basis. To ensure long-term accessibility, we are also exploring the creation of a mirror site to provide redundancy and safeguard data availability.

With these directions, MODOMICS is positioned to remain the central, community-driven resource for RNA modifications, interacting closely with other databases including Sci-ModoM and RNAcentral, supporting both fundamental research and applied developments in areas such as biotechnology, synthetic biology, and molecular medicine.

The rapid growth of high-throughput epitranscriptomic studies creates an urgent need for sustainable and secure storage of primary sequencing data, including sensitive human datasets. As repositories such as the European Genome-phenome Archive (EGA) no longer accept certain raw formats like POD5 from nanopore sequencing, long-term accessibility has become a major challenge. In line with the goals of the Human RNome Consortium, we call for coordinated international efforts to define best practices and establish infrastructures for preserving and sharing raw epitranscriptomic data.

## Supplementary Material

gkaf1284_Supplemental_File

## Data Availability

The data are accessible freely for research purposes at https://iimcb.genesilico.pl/modomics/.
