# Supplementary material for: MODOMICS: a database of RNA modifications and related information. 2025 update and 20th anniversary
Source: Nucleic Acids Res. 2025 Nov 24;54(D1):D219–25. doi: 10.1093/nar/gkaf1284 (PMC12807697; doi:10.1093/nar/gkaf1284)
Supplement: gkaf1284_Supplemental_File [file gkaf1284_supplemental_file.docx]

| \| **RNA type** \| \| --- \| | \| **Number of sequences** \| \| --- \| | \| **% of total** \| \| --- \| |
| --- | --- | --- | --- | --- | --- |
| \| mRNA \| \| --- \| | \| 39820 \| \| --- \| | \| 81.5 \| \| --- \| |
| \| unknown \| \| --- \| | \| 7062 \| \| --- \| | \| 14.5 \| \| --- \| |
| \| lncRNA \| \| --- \| | \| 1802 \| \| --- \| | \| 3.7 \| \| --- \| |
| \| pseudogene \| \| --- \| | \| 143 \| \| --- \| | \| 0.3 \| \| --- \| |
| \| snoRNA \| \| --- \| | \| 4 \| \| --- \| | <0.1 |
| \| miscRNA \| \| --- \| | \| 4 \| \| --- \| | <0.1 |
| \| snRNA \| \| --- \| | \| 1 \| \| --- \| | <0.1 |
| \| **Total** \| \| --- \| | \| **48836** \| \| --- \| | \| **100** \| \| --- \| |

Table 1: Overview of the newly added RNA sequences based on Sci-ModoM data.
